# Predictive Role of MicroRNAs in the Diagnosis and Management of Patients with Crohn’s Disease: A Clinician’s View

**DOI:** 10.3390/cells14181435

**Published:** 2025-09-13

**Authors:** Christian Prinz, Leonard Fehring

**Affiliations:** 1 Faculty of Health, School of Medicine, Witten/Herdecke University, 58455 Witten, Germany; 2 Helios University Hospital Wuppertal, Department of Gastroenterology, Witten/Herdecke University, 42283 Wuppertal, Germany; 3 Health Care Informatics, Faculty of Health, School of Medicine, Witten/Herdecke University, 58455 Witten, Germany

**Keywords:** Crohn’s disease, inflammation, ustekinumab, microRNA

## Abstract

Crohn’s disease (CD), also known as terminal ileitis, has been the focus of gastroenterological diagnostics and therapy for decades. Although significant therapeutic progress has been made in recent years, largely due to an improved understanding of the pathophysiology and evolving treatment strategies for Crohn’s disease, many new antibody-based therapies demonstrate clinical response rates of only 30–50%. Predictive biomarkers for differential therapeutic responses may therefore be critical for personalized treatment selection, but such markers have not yet been clinically validated for the majority of patients treated with prednisone or monoclonal antibodies targeting integrin pathways, TNF-α, or IL-23. In this review, the diagnostic potential of microRNA (miRNA) dysregulation in patients with Crohn’s disease is explored, emphasizing the potential utility of specific miRNA expression profiles in guiding targeted therapy. Notably, reduced expression of miR-29 is associated with planned treatment using ustekinumab (an IL-23 signaling inhibitor), elevated miR-23a levels in inflamed tissue may inform the use of TNF-α inhibitors, increased miR-155 expression is relevant for patients considered for JAK inhibitor therapy, and altered levels of miR-126 and miR-486 may support the selection of vedolizumab. Assessment of these dysregulated miRNAs—such as through comparative profiling in inflamed versus non-inflamed tissue from the same patients—could serve as a predictive biomarker panel to optimize individualized immunosuppressive treatment strategies in Crohn’s disease. We also examine the role of microRNAs in regulating TRP channels and their involvement in the mechanisms of action of selected complementary medicines.

## 1. Introduction

Crohn’s disease (CD), also known as terminal ileitis, is a chronic, relapsing inflammatory disorder affecting any part of the gastrointestinal tract, typified by discontinuous, transmural inflammation. It often leads to complications such as strictures, fistulas, and extra-intestinal manifestations. Accurate diagnosis is essential to optimize management and mitigate complications. Today, no single gold-standard test exists for diagnosing CD or guiding treatment decisions. Instead, CD is diagnosed using a multimodal approach that combines clinical evaluation with laboratory tests (including inflammatory markers and fecal calprotectin), endoscopic procedures with biopsies, and cross-sectional imaging such as MRI enterography or intestinal ultrasound, to confirm chronic intestinal inflammation and exclude alternative causes. Routine genetic or serological testing is not recommended for standard diagnostic work-up so far.

Until recently, first-line treatment for Crohn’s disease primarily involved broad T-cell-targeting drugs such as corticosteroids or azathioprine, both associated with considerable side effects, especially with long-term use. Systemic or topical steroids are still primarily employed in the acute phase. Approximately 70% of patients respond rapidly, but recurrence remains a major clinical challenge. For instance, an epidemiological study reported a relapse rate exceeding 50% within 12 months under combined therapy with glucocorticoids and 5-aminosalicylates (5-ASA) [1]. This high recurrence rate raises the question of whether traditional therapeutic goals in Crohn’s disease—including mucosal healing, stenosis prevention, and avoidance of surgery—can be sustainably achieved with “classical” therapies. The answer is likely no, and experienced clinicians are increasingly adopting novel therapeutic strategies that are both effective and durable.

In contrast, rheumatoid arthritis (RA) treatment with targeted antibody therapies is well established, resulting in symptom resolution, improved quality of life, and inhibition of joint destruction. As part of the “hit hard and early” strategy, potent and targeted immunosuppressants, including TNF-α blockers or interleukin-23 (IL-23) antagonists, have been introduced early in treatment for over 15 years. However, in countries like Germany, these therapies are largely restricted to outpatient settings due to costs and the lack of predictive biomarkers for therapeutic response, with success rates averaging 30–50% [1].

The development of neutralizing antibodies against TNF-α or IL-23 has introduced new treatment options for Crohn’s disease, similar to those in RA. Yet in practice, many patients fail to achieve durable responses, often due to economic limitations or treatment resistance. Thus, novel biomarkers are required prior to therapy initiation, since modern therapies are extremely cost-intensive, and predictive markers could potentially prevent ineffective treatments in up to 30–40% of patients. This review will present and discuss emerging tools based on specific therapy regimens.

## 2. The Role of microRNAs in Chronic Inflammatory Disorders and Cancer Diseases

Approximately 20 years ago, miRNAs were initially shown to play a key role in colorectal cancer (CRC). These small, non-coding RNA molecules, composed of 22–25 nucleotides, regulate mRNA translation by acting at the RNA-induced silencing complex (RISC). Humans express over 1000 known miRNAs, with many more presumed to exist. Recent studies have shown that miRNAs can influence programmed cell death by interacting with promoter regions of cell cycle regulators. Currently, around 1400 distinct miRNAs have been identified that bind to specific mRNA sequences, thereby inhibiting protein translation [2].

An increasing body of evidence suggests that microRNA (miRNA) dysregulation plays a causal or indicative role in many chronic inflammatory diseases of the gastrointestinal tract [3,4,5]. Differences in circulating miRNA levels—either in blood or in tissue samples—can serve as biomarkers for diagnosis, treatment response, or prognosis. Tissue-specific deregulation of miRNA expression has been reported in diseases such as multiple sclerosis and Crohn’s disease [6,7,8,9,10].

In recent years, circulating and fecal microRNAs (miRNAs) have gained attention as promising novel biomarkers for predicting therapeutic responses in patients with inflammatory bowel disease (IBD). Batra et al. reported significant alterations in the expression of seven miRNAs following treatment, distinguishing responders from non-responders in a small cohort of pediatric IBD patients receiving anti-TNF therapy [11]. However, a separate study investigating miRNA polymorphisms and their association with anti-TNF treatment response in Crohn’s disease (CD) found no significant correlations between the dysregulation of specific miRNAs (miR-146, miR-196a, miR-221, miR-224) and clinical outcomes in patients treated with anti-TNF monoclonal antibodies [12]. These conflicting findings underscore the need for further large-scale, well-controlled studies to validate the predictive value of miRNAs for treatment stratification in IBD.

## 3. MicroRNAs Dysregulation in Inactive (Quiescent) Crohn’s Disease

In a pioneering 2010 study, Fasseu et al. identified 14 and 23 miRNAs differentially expressed (0.001 < *p* < 0.05) in patients with inactive ulcerative colitis (UC) and inactive Crohn’s disease (CD), respectively [13]. Of these, eight miRNAs were commonly dysregulated in both diseases (miR-26a-5p, miR-29a-3p, miR-29b-5p, miR-30c-5p, miR-126-3p, miR-127-3p, miR-196a-5p, and miR-324-3p). Further analyses revealed that miR-26a-5p, miR-29b-5p, miR-126-3p, miR-127-3p, and miR-324-3p exhibited coordinated differential regulation in non-inflamed and inflamed colonic mucosa of patients with inflammatory bowel diseases. Other miRNAs, including miR-196b-5p, miR-199a-3p, miR-199b-5p, miR-320a-5p, miR-150-5p, and miR-223-3p, were differentially expressed between non-inflamed UC and CD tissue samples. Based on these findings, it has been proposed that miRNA dysregulation plays an important role in inflammation onset and relapse in IBD patients with quiescent mucosa [13].

## 4. MicroRNA Dysregulation in Active Crohn’s Disease

**Figure 1:**
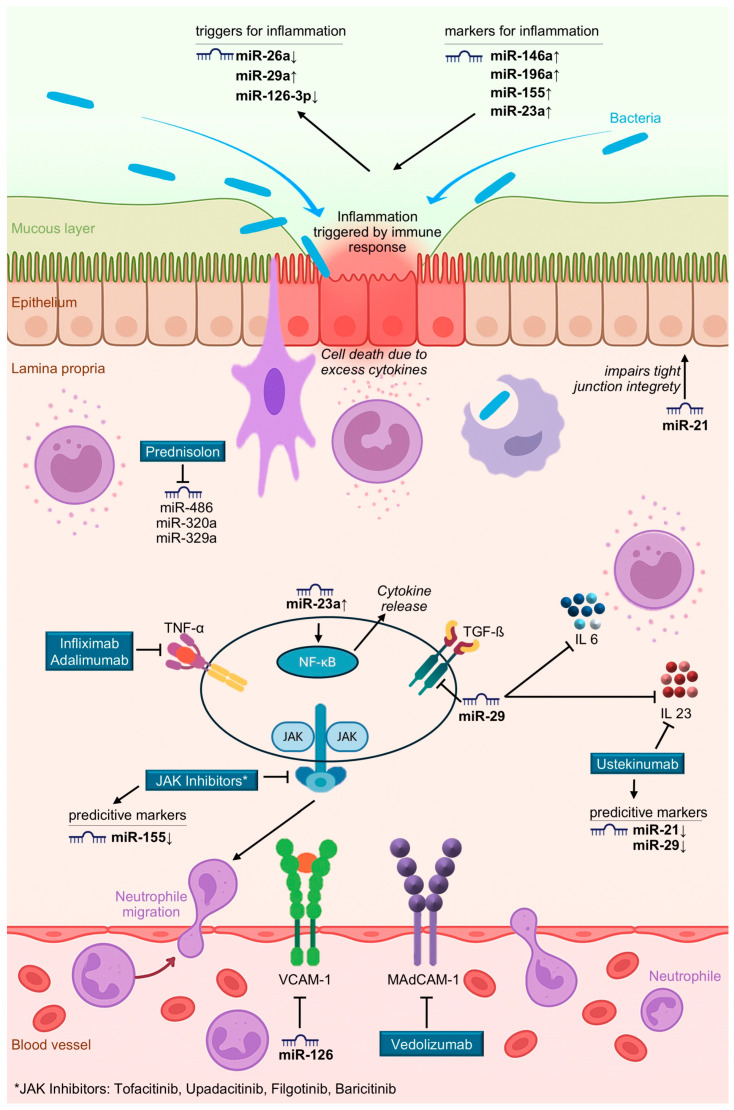
Illustration of selected MicroRNA dysregulation and Drug Targets in inflammatory bowel disease.

Initial evidence of altered miRNA expression in active IBD was reported by Wu et al., who found that patients with active UC exhibited elevated levels of eight miRNAs (miR-16, -21, -23a, -24, -29a, -126, -195, and Let-7f) and reduced levels of three miRNAs (miR-192, -375, and -422b) in intestinal mucosa compared to healthy individuals [14]. More recent work by de Sibia et al. identified ~15 upregulated and 6 downregulated miRNAs in active CD, including miR-16, -21, -23b, -31, -101, -106, -146, -191, -199a-5p, -206, -223, -340, -362–3p, and miR-375 [15]. An overview of the role of these microRNAs is given in Table 1 and Figure 1. Notably, miR-486 and miR-320a are particularly involved in traditional treatment responses with steroids (see Figure 1), as prednisone downregulates both [2]. Morilla et al. identified specific miRNAs associated with responses to corticosteroids, infliximab, and cyclosporine [16]. Cordes et al. reported that miR-320a levels were significantly increased in active IBD and strongly correlated with endoscopic disease activity, supporting its use as a non-invasive biomarker [17].

## 5. TRP Channels in Crohn’s Disease

Transient Receptor Potential (TRP) channels are a diverse family of ion channels located on cell membranes, where they regulate calcium, sodium, and other ion influx in response to stimuli such as temperature, mechanical stress, pH, and chemical signals. They are central to sensory processes, pain signaling, inflammation, and barrier integrity, making them relevant modulators to Crohn’s disease. TRP channels can be regulated by microRNAs (miRNAs), linking epigenetic mechanisms to channel function.

For example, the transient receptor potential vanilloid 1 (TRPV1) and ankyrin 1 (TRPA1) channels, predominantly expressed on capsaicin-sensitive sensory neurons, contribute to hyperalgesia and neurogenic inflammation [23,24]. In Crohn’s patients, TRPA1 promoter methylation is dysregulated, correlating with reduced pressure pain thresholds and suggesting epigenetic control of its expression [25]. While direct evidence in inflammatory bowel disease is limited, studies in irritable bowel syndrome show that miR-199 upregulates TRPV1, thereby enhancing visceral pain—an effect that is plausibly relevant to IBD [26].

TRPV4 is highly expressed in the intestinal epithelium and is frequently upregulated in IBD tissue, where it promotes barrier dysfunction, cytokine release, and altered motility [27,28,29]. Pharmacological blockade of TRPV4 alleviates experimental colitis [30]. Furthermore, TRPV4 is a validated target of miR-203; overexpression of this miRNA reduces TRPV4 levels and downstream signaling. Given TRPV4’s pro-inflammatory role in the gut, therapeutic delivery of miR-203 mimics represents a promising strategy to attenuate TRPV4-mediated pathology in Crohn’s disease [31].

## 6. Treatment with TNF-α Antibodies and the Role of Specific microRNAs

In Crohn’s disease, chronic inflammation is characterized by an accumulation of activated monocytes/macrophages that secrete TNF-α. Clinical studies have demonstrated the key role of TNF-α, and large international multicenter trials have assessed the efficacy of infliximab, a chimeric monoclonal antibody against TNF-α. Despite its effectiveness, infliximab induces remission in only ~30% of patients. In the ACCENT I trial, long-term response rates ranged between 17 and 22%, compared to 9% in the placebo group [32]. No predictive biomarkers for infliximab response have been validated.

Felwick et al. reported that miR-23a levels were significantly increased in Crohn’s epithelium relative to healthy tissue. miR-23a targets TNF-α inhibitor protein 3, leading to NF-κB activation, increased epithelial permeability, and elevated cytokine release, mimicking Crohn’s disease pathology [33].

## 7. Treatment with Vedolizumab and microRNA Dysregulation

Vedolizumab, a humanized integrin antagonist used in therapy of CD and ulcerative colitis (UC), shows a risk ratio (RR) of 2.01 (95% CI: 1.5–2.71) in three RCTs [34,35,36], achieving a significant therapy improvement in patients with CD. However, a meta-analysis showed no significant difference between Ustekinumab and Vedolizumab in patients refractory to TNF-α therapy [37]. miRNAs relevant to vedolizumab signaling include miR-126 (downregulated), affecting β-integrin activation. miR-126 also modulates VCAM-1, and its suppression mimics vedolizumab’s inhibition of mucosal addressin cell adhesion molecule-1 (MAdCAM-1) [38]. Harris et al. and Pathak et al. have demonstrated similar effects [39,40].

## 8. Treatment with Ustekinumab and microRNA Dysregulation

Ustekinumab targets IL-12/23 and demonstrates high efficacy in psoriasis and Crohn’s disease, particularly in TNF-refractory cases [41,42]. Using this new antibody, the UNITI-1 and UNITI-2 studies and meta-analyses report excellent efficacy in over 50% of CD patients, with RR values of 1.76 (95% CI: 1.4–2.22) and treatment success can be sustained for almost three years. However, dose escalation was required in over 80% of cases [43]. Ustekinumab shows the best long-term mucosal healing of all agents evaluated.

In this context, miRNAs such as miR-21 and miR-29 have been shown to regulate IL-17 and IL-23 expression, suggesting potential as predictive markers for ustekinumab response. miR-29 downregulates TGF-β, IL-6, and IL-23, mimicking ustekinumab’s effect [44,45]. miR-21 and miR-223 impair tight junction integrity, while miR-200 and miR-93 support it. miR-149 promotes a pathogenic microbiota shift.

Taken together, evaluation of these dysregulated miRNAs in inflamed versus healthy tissue could serve as a predictive biomarker panel to optimize individualized immunosuppressive treatment strategies in Crohn’s disease. In detail, maybe as a panel of investigated microRNA expression, reduced expression of miR-29 can favor a planned treatment using ustekinumab (an IL-23 signaling inhibitor), while elevated miR-23a levels in inflamed tissue may suggest the use of TNF-α inhibitors. Increased miR-155 expression could be relevant for patients considered for JAK inhibitor therapy, and altered levels of miR-126 and miR-486 may support the selection of vedolizumab. An overview summarizing the targets of mode of actions is provided in Figure 1 and Table 2, outlining targets and molecular pathways involved in the microRNA used as predictors.

## 9. Treatment with Complementary Medicine and microRNA Dysregulation

In recent years, traditional medicine-derived approaches have attracted growing interest as part of complementary and alternative medicine, with numerous studies investigating their potential role in Crohn’s disease. Among these, probiotics and microbiota modulation are the most extensively explored. Evidence suggests that the gut microbiota can reduce mucosal inflammation and improve epithelial barrier function. Strains such as *Escherichia coli* Nissle 1917 [46], as well as specific *Lactobacillus* and *Bifidobacterium* mixes [47,48], have been evaluated as potential therapeutic candidates. However, their clinical benefit has been demonstrated more consistently in ulcerative colitis than in Crohn’s. Importantly, microbiota can modulate host microRNA profiles in ways consistent with reduced inflammation and improved barrier integrity in experimental models, although human results remain variable and strain-specific [49]. For example, several probiotic strains reduce miR-155 expression in experimental colitis [50], an miRNA typically associated with pro-inflammatory pathways. In addition, some probiotics regulate miR-223 [49], which is linked to neutrophil activity and inflammasome regulation.

Another complementary approach is curcumin, the active compound in turmeric. Although clinical evidence is stronger for ulcerative colitis, mechanistic studies support its potential role in Crohn’s. Curcumin primarily acts by suppressing inflammatory pathways, particularly NF-κB signaling, thereby modulating immune responses [51]. Recent findings suggest that curcumin promotes intestinal epithelial renewal in part through downregulation of miR-195-3p, which otherwise inhibits epithelial proliferation and wound repair [52]. In cancer and inflammatory models, curcumin has also been shown to alter the expression of several key miRNAs, including miR-21, miR-34a, and miR-155, further highlighting its broad impact on inflammatory signaling pathways relevant to IBD [53].

Omega-3 polyunsaturated fatty acids (EPA and DHA) represent another widely studied intervention. These lipids influence cell membrane composition and eicosanoid balance. While mechanistic studies demonstrate modulation of inflammatory gene expression and epigenetic regulators, large high-quality clinical trials have shown that omega-3 supplementation is probably ineffective for maintaining remission in Crohn’s [54]. Nonetheless, omega-3 fatty acids appear to be safe, though they may cause diarrhea or upper gastrointestinal discomfort. In vitro and nutrimiromics studies show that EPA and DHA regulate several miRNAs involved in inflammatory responses of macrophages and epithelial cells, including miR-21, miR-146a, and miR-155, which are central players in NF-κB signaling and cytokine regulation [55].

Vitamin D has also been linked to IBD pathophysiology. Low vitamin D levels are associated with worse outcomes, while supplementation has been shown in some studies to reduce relapse risk and improve disease course [56]. Vitamin D signaling influences autophagy, barrier function, and innate immunity [56]. Correlative studies further suggest that vitamin D status is associated with distinct circulating miRNA profiles, and supplementation may alter levels of miRNAs involved in inflammatory and autophagy pathways, such as miR-142-3p [57].

Finally, several herbal compounds, such as *Boswellia serrata*, possess anti-inflammatory and anti-fibrotic properties demonstrated in animal colitis models and small human trials, often in combination with curcumin [58,59]. Evidence is emerging that these extracts can modulate miRNA expression in models of intestinal inflammation, generally reducing pro-inflammatory miRNA signatures and enhancing barrier markers. While specific effects are compound- and model-dependent, studies in other diseases have shown that Boswellia regulates miR-155 and to some extent other inflammation-related miRNAs [60,61]. To date, however, miRNA regulation by Boswellia has not been investigated in Crohn’s disease.

In summary, there is strong mechanistic plausibility that complementary interventions such as probiotics, curcumin, omega-3 fatty acids, vitamin D, and Boswellia may exert part of their therapeutic effects through modulation of host miRNA expression. This, in turn, could influence cytokine signaling, autophagy, tight junction integrity, and host-microbiota interactions. Nevertheless, most current evidence comes from cell culture systems and animal colitis models, with relatively few and often small human studies. Further well-designed clinical investigations are required before definitive conclusions can be drawn about the role of miRNA regulation in the efficacy of these complementary therapies in Crohn’s disease.

## 10. Strength of Evidence and Practical Takeaways

Although microRNAs are increasingly investigated as potential biomarkers for CD, their clinical application faces major challenges. One key limitation is their inherent instability, as they are rapidly degraded by RNases present in blood, stool, and other biological samples. This instability raises concerns about the reproducibility and reliability of circulating or fecal miRNA measurements as predictive biomarkers. To overcome these obstacles, approaches such as sample stabilization, encapsulation of miRNAs in extracellular vesicles, or normalization against stable reference molecules are being explored. Therefore, robust methodological advances are required before they can be translated into routine clinical practice. Firstly, one could analyze miRNA-associated proteins (such as Argonaute-bound complexes) [62]. Argonaute proteins bind the guide strand of the miRNA and mediate recognition of complementary sequences in target mRNAs. When miRNAs are bound to Argonaute proteins they are protected from RNase-mediated degradation, which enhances stability and makes them more reliable for measurement in blood or stool samples. Secondly, one could investigate exosomal miRNA. Exosomal microRNAs are packaged inside exosomes and secreted by cells into the extracellular environment. Finally downstream Cytokines could be a target of interest. Molecules like TNF-α, IL-6, IL-23, and IL-12 are already well-established in the inflammatory cascade of CD and can be quantified with standardized immunoassays (e.g., ELISA). In practice, a combined biomarker panel—integrating stable exosomal miRNAs, miRNA-protein complexes, and key cytokines—may be superior to any single biomarker type. Such a multimodal approach could improve both specificity and sensitivity in predicting therapeutic responses in CD, however so far no panel has been established yet.

Regardless of the method of measurement, several of the microRNAs discussed in this article appear mechanistically plausible and are supported by associative human data. Many have been reproducibly shown to be dysregulated in inflammatory bowel disease. However, variability in pre-analytical handling and platform-specific differences continue to limit analytical validity, which to date remains only moderate [63]. Similarly, the clinical validity of candidate panels remains uncertain. Existing studies have largely been conducted in small cohorts, leaving the current body of evidence insufficient to support routine clinical application.

As a result, no multi-miRNA panel has yet been prospectively validated to guide therapeutic decision-making in CD, and none are currently incorporated into clinical guidelines [1]. A comparable situation is observed in other gastrointestinal disorders. Panels have been proposed for early detection of colorectal cancer [64,65,66] and cholangiocarcinoma [67], but all remain at an early stage of validation. The investigated cohorts are typically modest in size, underscoring the need for large-scale, multicenter studies to establish both robustness and generalizability.

To move toward clinical implementation of miRNA panels in Crohn’s disease, three sequential steps are essential. First, assay protocols and cutoff values must be standardized, with harmonized approaches to sample collection, processing, and quantification, along with reproducible detection methods and clinically meaningful thresholds. Second, prospective multicenter validation is required. Candidate panels must be evaluated in large, geographically diverse cohorts of patients starting defined therapies, with baseline profiles correlated against standardized outcomes such as remission, mucosal healing, and biochemical response, and replicated across independent populations. Finally, clinical utility must be tested in impact studies. Randomized trials comparing panel-guided therapy with standard care should assess not only remission and mucosal healing but also safety, cost-effectiveness, and the avoidance of ineffective treatments.

Beyond their role as biomarkers, miRNAs also hold therapeutic potential in Crohn’s disease. By modulating the expression of key inflammatory mediators, they can suppress pathogenic pathways or enhance protective responses. For example, delivery of miRNA mimics may restore downregulated anti-inflammatory miRNAs such as miR-29 or miR-126, thereby reducing pro-inflammatory cytokine release and strengthening epithelial barrier function. Conversely, inhibition of upregulated pro-inflammatory miRNAs, such as miR-155 or miR-23a, using antagomirs or locked nucleic acid inhibitors, may attenuate NF-κB signaling and immune cell activation. Therapeutic approaches under investigation include systemic administration, tissue-targeted nanoparticles, and exosome-based delivery systems, offering a precision strategy to modulate disease-specific pathways. While preclinical studies in murine colitis models are encouraging [68], successful translation into clinical practice will require rigorous evaluation of safety, specificity, and long-term efficacy.

## 11. Conclusions

Over the past two decades, advances in our understanding of the molecular mechanisms underlying Crohn’s disease have paved the way for more targeted and effective therapeutic approaches. Despite these achievements, response rates to antibody-based therapies remain suboptimal, underscoring the urgent need for predictive biomarkers that can guide treatment decisions and minimize unnecessary exposure to ineffective and costly therapies.

Mounting evidence highlights the role of microRNAs as central regulators of immune signaling pathways and epithelial barrier function in Crohn’s disease. Specific dysregulation patterns—such as reduced miR-29 in patients likely to benefit from ustekinumab, elevated miR-23a in those eligible for TNF-α inhibitors, or altered expression of miR-126, miR-486, and miR-155 in the context of vedolizumab, corticosteroid, and JAK inhibitor therapy—suggest that miRNA profiling holds considerable promise as a tool for personalized treatment stratification.

While the preliminary findings are compelling, current data are largely derived from small cohorts or exploratory studies, and no miRNA-based biomarker panel has yet achieved clinical validation. Future research must therefore focus on large-scale, multicenter, and longitudinal studies to establish standardized assays, validate predictive accuracy, and assess the reproducibility of these findings across diverse patient populations. Integration of miRNA profiling with established clinical parameters may ultimately enable a precision medicine framework for Crohn’s disease, improving patient outcomes while optimizing healthcare resources.

## Figures and Tables

**Table 1:** General biomarkers of activity in patients with CD, serving as possible diagnostic markers.

microRNA	Targeting	Specific Role in CD and/or UC	Reference
miR-26a	significant role in regulating inflammatory responses., can reduce inflammatory responses by targeting genes like COX-2, leading to decreased production of inflammatory mediators like PGE2, TNF-α, IL-6, and IL-1β.	miR-26a: miR-26a deficiency or downregulation increases inflammation, as reported in some models of neuroinflammation and colitis.	[18]
miR-29a	influences macrophage behavior, promoting a shift towards anti-inflammatory M2-like macrophages while inhibiting pro-inflammatory M1-like macrophages.	miR-29a can also target and regulate various inflammatory pathways, such as the PI3K/AKT/mTOR pathway, which is involved in autophagy and inflammation, even in CD and UC.	[19]
miR-126–3p	role in inflammatory disorders by regulating vascular inflammation and endothelial cell function; targeting adhesion molecules like VCAM-1 and inhibiting the expression of pro-inflammatory cytokines.	Downregulation of miR-126–3p can contribute to increased inflammation, while its upregulation can have protective effects.	[20]
miR-146a	Combined detection of fecal calprotectin with miR-146a expression level improved the diagnostic sensitivity and the negative predictive value in differentiating IBD patients with active disease from those inactive.	One study identified a strong association of miR-146a rs2910164 GG genotype and G allele with IBD-increased susceptibility and activity in the Egyptian population.	[21]
miR-196a	Specifically, it downregulates the immunity-related GTPase family, M (IRGM) mRNA, which is associated with autophagy and can contribute to inflammation.	miR-196a has been found to be upregulated in the inflamed epithelium of Crohn’s Disease (CD) patients.	[22]

**Table 2:** Quintessence–Selective microRNA-biomarkers with a potential of predicting therapeutic response to antibody-based therapy in patients with Crohn’s Disease, based on elevated microRNA expression in inflamed tissue.

Substance of Planned Treatment	Target	Involved microRNA, Predictive Value of Specific Molecule	Mode of Action, Reference
Adalimumab, Infliximab	Human TNF-α antibody; Infliximab is a chimeric antibody directed against the cytokine TNF-α	miR-23a: miR-23a levels are significantly increased in inflamed Crohn’s epithelium relative to healthy tissue.	miR-23a targets TNF-α inhibitor protein 3, leading to NF-κB activation, increased epithelial permeability, and elevated cytokine release [33]
Vedolizumab	Humanized monoclonal antibody of the integrin antagonist class	miR-126: miR-126 levels are downregulated in patients with CD.	miR-126 affects β-integrin activation, and modulates VCAM-1; its suppression mimics vedolizumab’s inhibition of mucosal addressing the cell adhesion molecule-1 (MAdCAM-1) [38,39,40]
Ustekinumab	Monoclonal antibody selectively targeting and neutralizing the cytokines interleukin-12 (IL-12) and interleukin-23 (IL-23)	miR-21 and miR-29: both molecules regulate IL-17 and IL-23 expression, both of them being downregulated in CD, suggesting potential as predictive markers for ustekinumab response.	miR-29 downregulates TGF-β, IL-6, and IL-23 signaling, mimicking ustekinumab’s effect [44,45]. miR-21 also impairs tight junction integrity. [44,45]
Cortisone	Apoptosis of T-cells	miR-320a and miR-486 are upregulated in CD	miR-486 and miR-320a are particularly involved in traditional treatment responses, as prednisone downregulates both [2].
JAK Inhibitors	Affecting NF-κB signaling	miR-155, upregulated in CD but also in numerous other chronic inflammatory disorders	miR-155 is involved in several chronic inflammatory disorders, even in H. pylori infection [2].

## Data Availability

The datasets generated during and/or analyzed during the current study are available throughout the manuscript.
